# The changing patient profile: A retrospective study of trends in perioperative bleeding management and challenges in surgical care^✰^

**DOI:** 10.1016/j.sipas.2026.100345

**Published:** 2026-03-27

**Authors:** Pierre R. Tibi, Rory Tippit, Stephen M. Dierks, Natalia Peres Martinez

**Affiliations:** a Department of Cardiothoracic and Vascular Services, Yavapai Regional Medical Center, 1003 Willow Creek Rd, Prescott, AZ 86301, USA; b Medical Affairs Advanced Surgery, Worldwide Medical Affairs, Baxter Healthcare Corporation, 1 Baxter Parkway, Deerfield, IL 60015, USA

**Keywords:** Bleeding management, Charlson comorbidity index, Anticoagulant and antiplatelet use, Topical hemostatic agents, Patient complexity, Perioperative practice patterns

## Abstract

**Introduction:**

Medical innovations have extended patient lifespans while also increasing the clinical complexity associated with aging and chronic conditions. As patients live longer, surgeons may encounter an array of perioperative challenges including patients with multiple comorbidities. Consequently, surgical teams must manage bleeding risks while balancing potential thromboembolic complications.

**Material and Methods:**

This retrospective observational study evaluated trends in perioperative bleeding management and associated surgical care challenges over 22 years, utilizing data from the Premier Perspective Hospital Database. The analysis included 13,358,404 adult surgical patients, examining patient complexity via Charlson Comorbidity Index (CCI), preoperative anticoagulant/antiplatelet usage, and topical hemostatic agent (THA) utilization.

**Results:**

Patient complexity, as indicated by CCI, significantly increased annually across all surgical types, with the greatest increase in cardiovascular surgery (0.029/year). Overall preoperative use of anticoagulants and antiplatelets rose by 0.5 % per year, with marked variations across surgical cohorts. Notably, general surgery exhibited the highest annual increase (2.0 %), while knee and hip replacements showed a decline in anticoagulant/antiplatelet use. Overall, use of THA strategies increased by 0.56 % annually, particularly in neurosurgery, spinal, and cardiovascular procedures. Multivariate analysis demonstrated significant associations between patient complexity, anticoagulant/antiplatelet use, and higher use of THA strategies across various surgical disciplines. Elective surgeries were more likely to be associated with THA charges than emergent or urgent procedures.

**Conclusions:**

This large retrospective analysis describes evolving patient complexity, antithrombotic medication use, and associated use of THA strategies across surgical specialties over two decades. These findings characterize practice patterns and may inform future hypothesis-driven studies evaluating the clinical role of THAs in different surgical contexts.

## Highlights

• Patient comorbidity has increased across surgical specialties.
• Anticoagulant and antiplatelet use has increased across surgical specialties.
• The use of topical hemostatic agent strategies increased with specialty-specific variations.
• Findings describe utilization trends and support future-hypothesis-driven research.

## 1 Introduction

As a result of innovative medical interventions, the elderly population and lifespans of individuals with chronic conditions in modern countries are growing [[1], [2], [3]]. As patients live longer, the comorbidity burden in those undergoing surgery has continued to expand. It has been reported that multiple comorbidities or “multimorbidity” is already present in >60 % of those aged ≥65 years among the general population [4]. Studies also highlight the increasing prevalence of multimorbidity among younger populations contributing to complexities in healthcare [5,6].

Evaluation of comorbidities has been used to predict a variety of outcomes including functional status, quality of life, complications, readmissions, and health care utilization [7]. Studies have highlighted the importance of the Charlson Comorbidity Index (CCI) as a critical tool for measuring patient complexity and predicting long-term outcomes [7,8].

With increasing life expectancy and a higher prevalence of chronic diseases, there has also been a marked rise in the use of anticoagulant and antiplatelet therapies, reflecting the older age and greater clinical complexity of patients presenting for surgery [9]. Therefore, the management of anticoagulated patients in the perioperative setting demands a nuanced approach to balance the need for hemostasis with the prevention of thromboembolic events.

To address bleeding challenges in complex, high-risk surgical patients, topical hemostatic agents (THAs), including passive and active types, have become increasingly popular [8,10]. Prior studies have reported associations between active hemostatic products and reduced bleeding-related complications, transfusions, and associated costs in major surgeries [11,12].

Given the evolving nature of patient profiles and increasing use of antithrombotic medications, there is a critical need to re-evaluate how these factors affect perioperative hemostatic management. This retrospective study was designed to analyze trends in patient complexity, medication use, and the corresponding strategies for managing perioperative bleeding in a modern surgical population.

## 2 Material and methods

### 2.1 Study design

This study was a retrospective observational analysis based on surgical inpatient data from the Premier Perspective Hospital Database. This proprietary hospital database captures complete patient billing, hospital cost, and coding histories from >1100 healthcare facilities throughout the United States (US). Data include a date-stamped log of all billed items including procedures, diagnoses, medications, laboratory, diagnostic/therapeutic services, costs, drugs, and medical devices received at the individual patient level during hospitalization. All data used in the analysis were de-identified and did not meet the criteria for human subject research. Thus, an exemption was granted by a central Institutional Review Board (IRB) based on criteria specified in 45 CFR 46.101(b)(4), as this research involved the collection and study of existing data. Additionally, the data were recorded in an anonymous manner such that subjects could not be identified directly or through identifiers linked to them.

### 2.2 Study population

Surgical patients aged 18 years or older discharged between January 2000 and December 2021 were included in the analysis. Patients were excluded if they had incomplete demographics, if their surgery occurred >90 days after admission, if they had multiple types of surgical procedures within their admission, or if they had multiple surgical procedures across different days of their admission. Surgical procedures were classified into the following eight groups: cardiovascular/cardiothoracic (coronary artery bypass graft [CABG], cardiac valve procedures, and lung resection); general surgery (colectomy, thyroidectomy, pancreatectomy, liver resection, and gastrectomy); gynecologic (hysterectomy); knee and hip replacements; neurosurgery; solid organ and hepatopancreatobiliary (HPB); spine; and urologic.

### 2.3 Variables

The following patient characteristics were analyzed: age at the time of admission (years); gender: male, female (unknown was excluded); race: white, black, other; surgical procedure type; insurance payor type: Medicare, Medicaid, private pay, uninsured or unknown; admission type: elective, emergent, urgent; use of anticoagulants or antiplatelets immediately before the surgical procedure; CCI; use of THAs during surgical procedure on same day as surgical procedure. The following hospital characteristics were analyzed: teaching hospital status: yes, no; location: urban, rural; US geographic region: East, Midwest, South, West; bed count: 0–199, 200–299, 300–499, 500+.

Anticoagulant/antiplatelet and THA use were identified from hospital billing charges. Each patient was classified as having received or not having received anticoagulants/antiplatelets and THAs. The Premier database captures products that were charged but does not confirm actual intraoperative application, timing, dose, or indication. For the purposes of this descriptive analysis, the presence of a charge was used as a proxy for potential product use. However, particularly for THAs, product charging or opening does not guarantee that the product was ultimately applied during the procedure. THA strategy was defined as active only, passive only, passive and active, or no passive or active.

Patient complexity was analyzed using the CCI from ICD-10 and ICD-9 diagnosis codes present during admission. For each patient, CCI was determined using a composite score calculated from the following clinical conditions and corresponding scores. Score +1: cerebrovascular disease, chronic liver disease, chronic pulmonary disease, congestive heart failure, connective tissue disease, dementia, diabetes, myocardial infarction, peripheral vascular disease, ulcer; Score +2: diabetes with end organ damage, hemiplegia, leukemia, lymphoma, moderate or severe kidney disease, paraplegia, tumor; Score +3: moderate or severe liver disease; Score +6: acquired immune deficiency syndrome (AIDS), malignant tumor, metastasis.

### 2.4 Statistical analysis

Patient and hospital characteristics were summarized descriptively overall and by study cohort. Continuous variables were summarized using descriptive statistics (n, mean, standard deviation, median, minimum, and maximum), and categorical variables were presented as counts and percentages.

Trends of averages and percentages for CCI, anticoagulant/antiplatelet usage, and THA usage were calculated annually over the 22-year period. Best fit trendlines were calculated using linear regressions for the aggregated sample of all surgical patients and for each surgical cohort. Years 2000 to 2021 were coded as 0 to 21 and significance was set to α=0.050. Analyses were performed using Stata version 17.0 [13].

Multinomial logistic regressions were used to analyze patient and hospital characteristics that were associated with the use of each THA strategy. To determine whether patient comorbidities and the use of anticoagulants/antiplatelets were associated with the various THA strategies, CCI and anticoagulants/antiplatelets usage were included as independent variables. To determine whether surgical type was associated with the use of the various THA strategies, the surgical cohort was included as a dummy variable. Results were reported as odds ratios and 95 % confidence intervals. Analyses were performed using SAS version 9.4 (SAS Institute Inc, Cary, NC).

## 3 Results

### 3.1 Study population

A total of 13,358,404 surgical patients in 1125 hospital-years met inclusion criteria and were included in the analysis. Specific characteristics of the study population are provided in Table 1. Among all 1125 hospital-years, 65 reported no THA usage (passive or active) for any of the 166,774 surgical patients in those 65 hospital-years. In the cardiovascular cohort of 1019 hospital-years, 111 hospital-years never used a THA for any of their cardiovascular surgical patients prior to surgery.

**Table 1:** Characteristics of the study population.

Characteristic	Facilities	Patients
Total Sample	1125	13,358,404
Facility Level: No Hemostatic Agent	65	166,774
Facility Level: Passive Only Hemostatic Agent	62	35,999
Facility Level: Passive/Active Hemostatic Agent	952	12,815,160
Facility Level: Active Only Hemostatic Agent	46	340,471
Facility Level (No Variance): No Hemostatic Agent	65	166,774
Total Facilities with Variance	1060	13,191,630
Total facilities with no Variance	65	166,774

### 3.2 Patient complexity

On average, mean CCI increased year over year overall and for each surgical cohort examined (**Figure S1**; Table 2; *p* < 0.001). However, the degree to which CCI increased varied considerably depending on the cohort ranging from 0.004 per year for General surgery (*p* < 0.001) to 0.029 per year for Cardiac/Vascular surgery. The increases in trends were significant at the *p* < 0.001 level for all cohorts.

**Table 2:** Mean charlson comorbidity index increases year over year for each surgical cohort.

Surgery	Slope	R^2^
All	0.012	75.1 %
Cardiovascular	0.029	95.3 %
General	0.004	47.9 %
Knee/Hip	0.014	71.8 %
Neuro	0.015	89.8 %
Gynecologic	0.016	94.8 %
Solid/Hepatopancreatobiliary	0.022	65.8 %
Spinal	0.017	66.8 %
Urology	0.019	91.0 %

### 3.3 Anticoagulants/Antiplatelets analysis

Preoperative anticoagulants/antiplatelets use increased on average by 0.5 % per year in the overall surgical sample (**Figure S2;**Table 3; *p* < 0.001;); however, change in use varied considerably across the different surgical cohorts. The knee and hip replacement group was the only cohort to have a decline in the use of anticoagulants and antiplatelets**.** There was no discernable change in the use of anticoagulants/antiplatelets over time for the neurosurgical cohort (slope = 0.000; *p* = 0.752). All other cohorts saw a significant increase with the largest impact in the general surgery cohort where use increased on average by 2.0 % each year (*p* < 0.001).

**Table 3:** Anticoagulant/antiplatelet use year over year for each surgical cohort.

Surgery	Slope	R^2^
All	0.005	13.8 %
Cardiovascular	0.015	56.2 %
General	0.020	89.2 %
Knee/Hip	−0.019	58.8 %
Neuro	0.000	0.5 %
Gynecologic	0.010	98.1 %
Solid/Hepatopancreatobiliary	0.016	81.4 %
Spinal	0.004	53.2 %
Urology	0.014	96.4 %

### 3.4 Topical hemostatic agent analysis

Overall, the percentage of THA charges for the entire surgical sample was 30.1 % (Table 4). From 2017–2021 the surgical types with the highest overall use of THAs were, in order: neurosurgery, spinal, cardiovascular, solid organ/HPB, and urology. Those same five surgical types had the highest use of active only and passive plus active THA strategies. The surgical types that used the most passive-only THAs from 2017 to 2021 were: solid organ/HPB, urology, cardiovascular, gynecologic, and general.

**Table 4:** Percentage of use topical hemostatic agent strategies for the surgical sample.

	Overall THA Use		Passive Only Use		Passive + Active Use		Active Only Use	
Surgery	Last 5 yrs	Trend	Last 5 yrs	Trend	Last 5 yrs	Trend	Last 5 yrs	Trend
All	30.1 %	0.56***	8.7 %	0.03	11.1 %	0.09*	10.3 %	0.44***
CV	54.0 %	1.61***	20.7 %	0.36	21.2 %	0.72***	12.1 %	0.53***
General	15.2 %	0.58***	7.0 %	0.16***	1.4 %	0.06***	6.7 %	0.36***
Knee	4.2 %	0.11	1.6 %	0.06***	0.2 %	0.01	2.3 %	0.05
Neuro	76.7 %	1.61***	6.5 %	−0.41***	54.7 %	1.40***	15.4 %	0.61***
Gyn	25.2 %	1.22***	15.1 %	0.61***	2.2 %	0.13***	7.9 %	0.49***
Solid HPB	50.4 %	1.49***	22.6 %	0.35**	16.6 %	0.70***	11.2 %	0.44***
Spinal	73.9 %	0.73**	4.9 %	−0.56***	35.9 %	−0.13	33.1 %	1.41***
Urology	45.8 %	1.95***	21.2 %	0.70***	14.1 %	0.73***	10.4 %	0.52***

Abbreviations: CV = cardiovascular; Gyn = gynecologic; HPB = hepatopancreatobiliary; THA = topical hemostatic agents; yrs = years.

*p < 0.05; **p < 0.01; ***p < 0.001.

**Fig. 1:**
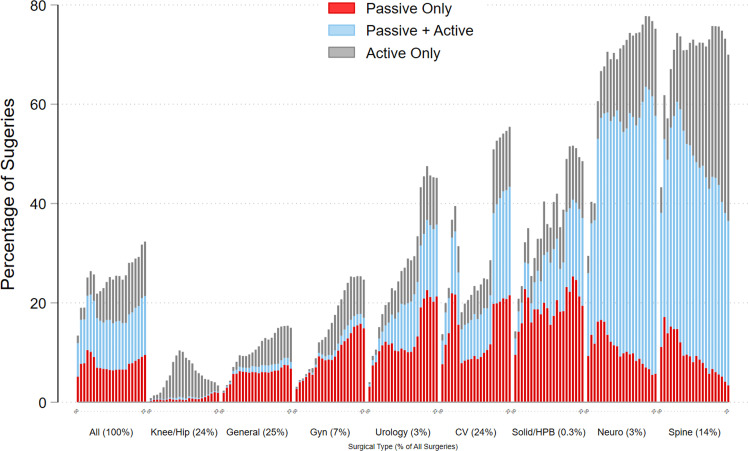
Use of each hemostatic strategy (passive hemostatic agents only; passive and active hemostatic agents; active only hemostatic agents. Abbreviations: CV = cardiovascular; HPB = hepatopancreatobiliary; neuro = neurosurgery.

The trend of using THA strategies for all surgical types increased from 2000 to 2021 where on average use of THAs increased by 0.56 % each year (slope = 0.56, *p* < 0.001). However, the trend in use of THAs varied considerably by the type of surgery and the THA strategy. For example, within spinal surgery, the use of all THAs increased, yet this change was driven by an increase in the use of active-only THAs which outpaced the decline in the use of passive-only THAs (Fig. 1). Cardiovascular, general, gynecologic, solid organ/HPB, and urology saw significant increases in each of the three THA strategies.

### 3.5 Multinomial logistic regressions

Overall, the likelihood of THA usage increased year over year across all THA strategies compared to no THA (Table 5). CCI was positively associated with an increased likelihood of using any of the THA strategies. The use of anticoagulants/antiplatelets was positively associated with an increased use of passive, passive plus active, and active THAs. Compared to elective surgeries, emergent/urgent surgeries were less likely to use any THA.

**Table 5:** Multinomial logistic regression with adjusted odds ratio and baseline use of topical hemostatic agent strategies.

	(1) Passive only		(2) Passive plus active		(3) Active only	
Variables	Adjusted OR	95 % CI	Adjusted OR	95 % CI	Adjusted OR	95 % CI
Annual trend	1.029***	(1.029 - 1.030)	1.054***	(1.053 - 1.054)	1.095***	(1.095 - 1.096)
Specialty (Baseline = General)						
Cardiovascular	2.339***	(2.323 - 2.354)	2.888***	(2.856 - 2.921)	1.730***	(1.716 - 1.743)
Knee	0.105***	(0.104 - 0.107)	0.050***	(0.049 - 0.052)	0.790***	(0.784 - 0.796)
Neurosurgery	5.801***	(5.723 - 5.880)	53.658***	(52.936 - 54.390)	14.033***	(13.854 - 14.215)
Gynecologic	1.531***	(1.518 - 1.545)	0.335***	(0.328 - 0.342)	1.133***	(1.120 - 1.145)
Solid organ/hepatopancreatobiliary	4.393***	(4.277 - 4.513)	3.966***	(3.833 - 4.105)	2.590***	(2.495 - 2.688)
Spinal	4.318***	(4.284 - 4.351)	34.702***	(34.315 - 35.092)	17.558***	(17.429 - 17.689)
Urology	2.633***	(2.606 - 2.659)	2.846***	(2.805 - 2.888)	1.948***	(1.924 - 1.973)
Patient characteristics						
Anticoagulant / antiplatelet	1.185***	(1.178 - 1.191)	1.710***	(1.700 - 1.720)	1.523***	(1.515 - 1.531)
Charlson Comorbidity Index	1.070***	(1.068 - 1.073)	1.112***	(1.110 - 1.115)	1.051***	(1.049 - 1.054)
Age	1.001***	(1.001 - 1.001)	1.000***	(0.999 - 1.000)	0.999***	(0.999 - 0.999)
Male Gender	1.053***	(1.048 - 1.058)	1.006*	(1.001 - 1.010)	0.982***	(0.978 - 0.987)
Unknown Gender	0.672*	(0.504 - 0.896)	0.327***	(0.236 - 0.453)	1.100	(0.883 - 1.371)
Race (Baseline = White)						
Black	1.004	(0.997 - 1.011)	0.946***	(0.938 - 0.953)	0.954***	(0.947 - 0.961)
Other/Unknown	0.885***	(0.880 - 0.891)	0.883***	(0.877 - 0.888)	0.949***	(0.943 - 0.955)
Admission (Baseline = Elective)						
Emergency	0.518***	(0.515 - 0.521)	0.373***	(0.371 - 0.375)	0.376***	(0.373 - 0.378)
Urgent	0.605***	(0.601 - 0.609)	0.518***	(0.515 - 0.522)	0.508***	(0.504 - 0.512)
Other/Unknown	0.776***	(0.762 - 0.790)	0.747***	(0.735 - 0.759)	0.589***	(0.578 - 0.601)
Payor (Baseline = Private)						
Medicare	1.008*	(1.002 - 1.014)	0.922***	(0.917 - 0.928)	0.928***	(0.922 - 0.933)
Medicaid	1.036***	(1.027 - 1.044)	0.912***	(0.903 - 0.920)	0.880***	(0.873 - 0.888)
Uninsured/Unknown	1.044***	(1.035 - 1.052)	0.937***	(0.930 - 0.945)	0.873***	(0.866 - 0.881)
Hospital characteristics						
Region = Midwest	0.993	(0.986 - 1.000)	1.305***	(1.295 - 1.315)	1.088***	(1.080 - 1.096)
Region = South	1.266***	(1.258 - 1.274)	1.880***	(1.867 - 1.893)	1.364***	(1.355 - 1.373)
Region = West	0.818***	(0.811 - 0.825)	1.272***	(1.261 - 1.283)	1.567***	(1.555 - 1.579)
Urban	1.133***	(1.124 - 1.142)	1.066***	(1.057 - 1.075)	1.048***	(1.040 - 1.056)
Teaching	1.021***	(1.016 - 1.026)	1.119***	(1.113 - 1.125)	1.035***	(1.030 - 1.040)
Bed Count	0.974***	(0.973 - 0.976)	0.968***	(0.966 - 0.970)	0.994***	(0.993 - 0.996)

Abbreviations: CI = confidence interval; OR = odds ratios.

*p < 0.05; **p < 0.01; ***p < 0.001.

## 4 Discussion

This study describes long-term trends in perioperative bleeding management within the context of evolving patient complexity across multiple surgical specialties. The findings underscore consistent increases in comorbidity burden over time, accompanied by rising preoperative anticoagulant and antiplatelet medication charges and increasing THA charges, with substantial heterogeneity by surgical cohort. Collectively, these findings characterize contemporary perioperative practice patterns and highlight important differences across specialties

### 4.1 Increased patient complexity and antithrombotic drug use

It has been estimated that 23 % of the total global disease burden disorders are in people aged 60 years or more [14]. Just under a third of the disease burden in older adults is due to cardiovascular diseases, followed by malignant neoplasms, chronic respiratory diseases, musculoskeletal diseases, and neurological and mental disorders [15]. Multimorbidity is also increasingly prevalent among young and middle-aged adults, with common conditions including hypertension, dyslipidemia, depression, and diabetes [5]. These demographic shifts are reflected in surgical populations, where increasing comorbidity burden has been associated with higher perioperative risk.

Aging is associated with alterations in the coagulation system and increased prevalence of cardiovascular and thromboembolic disease, contributing to widespread use of antiplatelet and anticoagulant therapies [16]. Taken together, these trends contribute to greater surgical complexity and heightened bleeding risk [3].

Consistent with these observations, mean CCI increased significantly year over year in our study, with the largest increases observed among cardiovascular and neurosurgical patients. These more pronounced increases in CCI in these cohorts align with the complex health profiles typical in these patient populations [[17], [18], [19]].

Preoperative anticoagulant and antiplatelet use increased by approximately 0.5 % per year across the overall surgical sample, reflecting broader trends in medical practice. While these medications are essential for preventing thromboembolic events and improving long-term outcomes, they pose challenges for intraoperative bleeding management [8]. The mechanisms of action of antithrombotic agents may impair hemostatic efficacy and increase bleeding risk, underscoring the importance of effective perioperative bleeding management strategies [8,20].

Interestingly, anticoagulant and antiplatelet use remained stable over time in neurosurgical patients. This pattern may reflect the inherently high bleeding risk associated with neurosurgical procedures [21], where even small hemorrhages can have catastrophic consequences due to the delicate vascular structures of the brain and spinal cord [22]. Longstanding clinical guidance emphasizing minimization of hemorrhagic complications may contribute to a consistently conservative approach in this population [23].

The decrease in anticoagulant and antiplatelet use before knee and hip surgeries may reflect the influence of evolving perioperative management strategies tailored to these procedures, including stricter adherence to perioperative protocols, emphasizing the suspension or bridging of anticoagulant and antiplatelet therapies to minimize intraoperative bleeding.

### 4.2 Topical hemostatic agent use in elective vs. emergency surgeries

The increasing complexity of patients necessitates the use of more advanced hemostatic strategies, including the use of THAs, particularly in bleeding involving sensitive structures or in patients with hemostatic abnormalities. In our study, higher frequencies of THA charges were observed in cardiovascular and neurosurgical procedures, where bleeding risk is high [8,16]. This data highlights the need for careful preoperative planning, particularly in patients on antithrombotic therapy, to mitigate the risk of excessive bleeding during surgery.

A notable finding was the lower frequency of THA strategies in emergency and urgent surgeries compared with elective procedures. Emergency settings often require rapid mechanical control of bleeding, which may limit opportunities for adjunctive topical interventions [24,25]. In addition, limited data and lack of comprehensive guidelines regarding THAs in emergency surgery may contribute to variability and hesitancy in application [26,27]. Further research is needed to clarify the role of THAs in emergency surgery scenarios.

### 4.3 Decreased use of topical hemostatic agents in orthopedic surgeries

Our analysis revealed a declining trend in the use of THAs in orthopedic surgeries, particularly in knee and hip replacements. This pattern may reflect widespread adoption of alternative blood conservation strategies in orthopedic surgery, including perioperative antithrombotic management guided by clinical practice recommendations [28]. Preoperative strategies to reduce blood loss in orthopedic surgery include halting anti-inflammatory and blood-thinning medications [29]. Intraoperative techniques such as hypotensive anesthesia, tourniquet use, topical antifibrinolytics, and routine administration of tranexamic acid may further reduce reliance on THAs. Although THAs are used in orthopedic surgery, they remain less studied in this specialty than in others

### 4.4 Variability in topical hemostatic agent use across specialties

THAs have become increasingly available and are often effective in achieving local hemostasis within minutes [30]. These products can be broadly categorized as passive agents, which rely on contact activation and platelet aggregation, and active agents, which supply exogenous coagulation factors and act independently of the patient’s coagulation status [8,10,25]. Despite expanded availability, substantial variability in THA strategies persist across different surgical specialties [8,30,31]. Neurosurgery, cardiovascular surgery, and HPB surgery demonstrate particularly high use of THA strategies, particularly active agents such as thrombin-based products [8,32].

Iannitti et al. [8] reported that, among inpatient surgical populations, use of passive hemostatic products alone was associated with higher rates of bleeding-related complications and increased hospital costs compared with active hemostatic approaches. While these findings suggest that passive products alone may be insufficient in certain high-risk surgical contexts, comparative effectiveness data remain limited. In the absence of robust comparative studies and specialty-specific guidelines, THA selection may reflect physician preference and institutional practice rather than standardized criteria [30,31].

### 4.5 Limitations

This study has several important limitations. First, it is a retrospective observational analysis based on administrative data from the Premier database. Charges were used as proxies for product use and do not confirm actual intraoperative application, timing, dose, or indication. This limitation is particularly relevant for THAs as product charging or opening does not guarantee that the product was ultimately used. Consequently, THA utilization may be systemically overestimated.

Second, the study does not include linked clinical outcomes such as bleeding complications, transfusions, reoperations, thromboembolic events, or mortality, precluding assessment of effectiveness, safety, or comparative performance of THA strategies. Third, observed associations represent temporal relationships rather than causal effects. Finally, increasing CCI over time may partly reflect secular changes in coding practices, including transition from ICD-9 to ICD-10 and improved documentation, in addition to changes in underlying patient health.

### 4.6 Implications for future research

The year-over-year increase in the CCI among surgical patients reflects a growing trend toward treating older, more medically complex populations. The widespread use of anticoagulants and antiplatelets across surgical cohorts mirrors their growing role in managing thromboembolic risks in an aging population. The complexity of surgical patients, combined with the widespread use of antithrombotic medications, necessitates a more strategic approach to perioperative bleeding management.

This analysis does not evaluate clinical outcomes such as bleeding complications, transfusion requirements, reoperations, thromboembolic events, or mortality, and therefore cannot determine whether increased use of THAs is beneficial, neutral, or excessive, nor assess comparative effectiveness between THA strategies. In addition, intraoperative timing, dose, indication, and surgeon intent for THAs are not captured within administrative billing data.

However, by characterizing large-scale, long-term patterns in patient complexity, antithrombotic medication exposure, and use of THA strategies across diverse surgical specialties, this study provides an important descriptive framework for future investigation. The observed specialty-specific trends and associations identify clinical contexts in which prospective, outcomes-based studies may be most informative and support the development of targeted hypotheses regarding when, where, and for whom THAs may offer the greatest value.

## 5 . Conclusions

This study provides a large-scale descriptive assessment of long-term trends in patient complexity, antithrombotic medication use, and THAs across multiple surgical specialties. Increasing CCI scores and rising antithrombotic exposure were temporally associated with greater THA charges, with substantial heterogeneity across procedure types. While this observational analysis, based on administrative data, does not establish causality or evaluate clinical outcomes, it offers a comprehensive, contemporary view of perioperative bleeding management patterns in routine practice. Collectively, these findings establish an important descriptive foundation that can inform future prospective, outcomes-based studies aimed at better defining the role of THAs in modern surgical care.

## Funding and role of the sponsor

Funding: This work was supported by Baxter Healthcare. Baxter Healthcare was involved in the study design; data collection, analysis, and interpretation; writing of the report; and the decision to submit the article for publication.

## CRediT authorship contribution statement

**Pierre R. Tibi:** Writing – review & editing, Writing – original draft, Validation, Supervision, Conceptualization. **Rory Tippit:** Writing – review & editing, Validation, Supervision. **Stephen M. Dierks:** Writing – review & editing, Validation, Supervision, Methodology, Formal analysis, Data curation, Conceptualization. **Natalia Peres Martinez:** Writing – review & editing, Validation, Supervision.

## Declaration of competing interest

The authors declare the following financial interests/personal relationships which may be considered as potential competing interests:

Pierre Tibi reports a relationship with Baxter Healthcare that includes: non-financial support. Rory Tippit reports a relationship with Baxter Healthcare that includes: employment. Stephen M. Dierks reports a relationship with Baxter Healthcare that includes: employment. I Natalia Peres Martinez reports a relationship with Baxter Healthcare that includes: employment. The authors declare that there are no other known competing financial interests or personal relationships that could have appeared to influence the work reported in this paper.

## Appendix Supplementary materials

Image, application 1
