# Supplementary material for: The changing patient profile: A retrospective study of trends in perioperative bleeding management and challenges in surgical care
Source: Surg Pract Sci. 2026 Mar 27;25:100345. doi: 10.1016/j.sipas.2026.100345 (PMC13084462; doi:10.1016/j.sipas.2026.100345)
Supplement: Supplementary file 1 [file mmc1.docx]

**SUPPLEMENTARY MATERIAL**

**
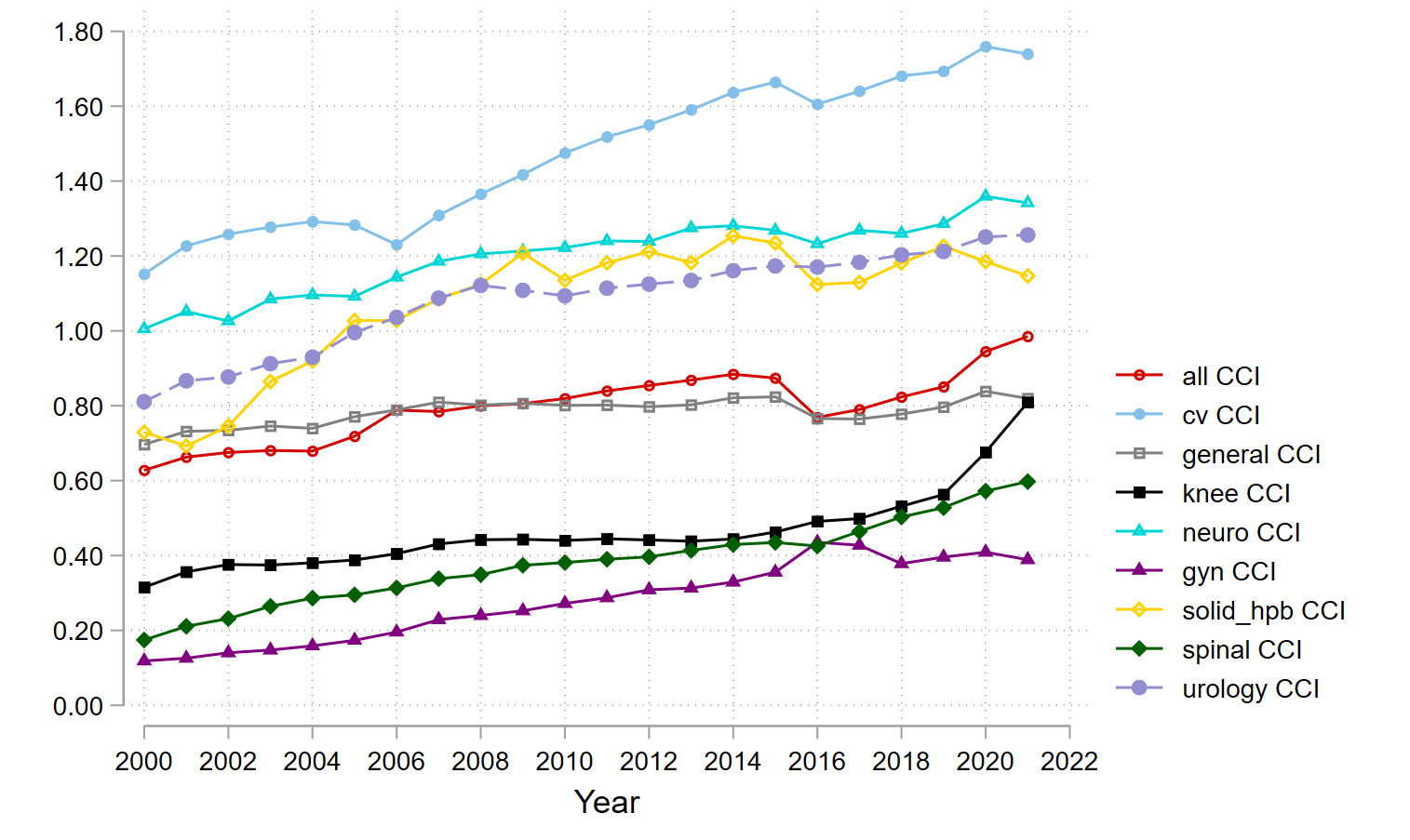
**

**Figure S1:** Mean Charlson Comorbidity Index (CCI) increases over time. Abbreviations: CV = cardiovascular; gyn = gynecologic; HPB = hepatopancreatobiliary; neuro = neurosurgery.

**
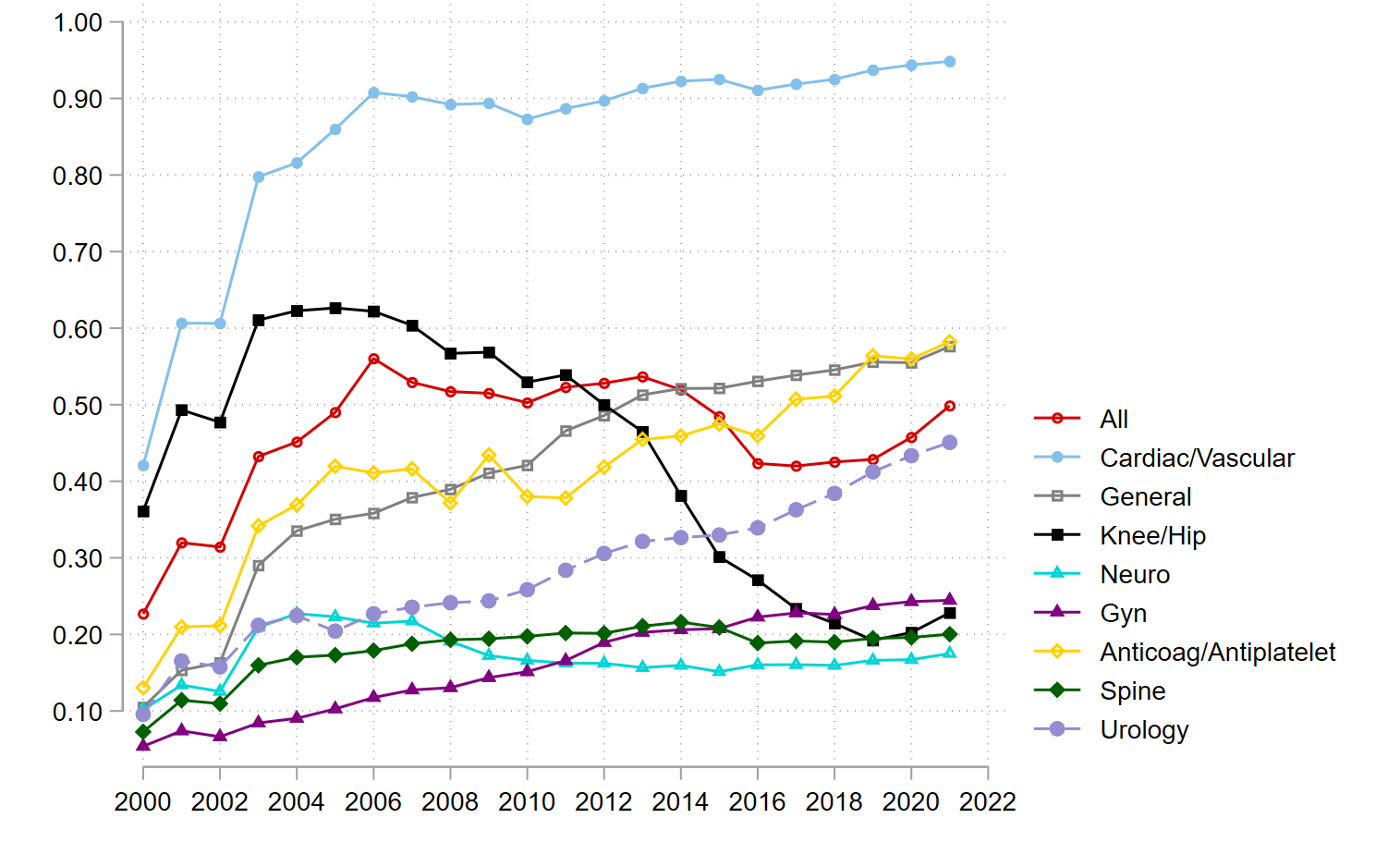
**

**Figure S2:** Percentage of anticoagulant or antiplatelet use over time. Abbreviations: CV = cardiovascular; HPB = hepatopancreatobiliary; neuro = neurosurgery.
